# An Effective Mechanism for the Early Detection and Containment of Healthcare Worker Infections in the Setting of the COVID-19 Pandemic: A Systematic Review and Meta-Synthesis

**DOI:** 10.3390/ijerph19105943

**Published:** 2022-05-13

**Authors:** Yueli Mei, Xiuyun Guo, Zhihao Chen, Yingzhi Chen

**Affiliations:** 1 School of Political Science and Public Administration, East China University of Political Science and Law, Shanghai 201620, China; mia_meiyueli@sjtu.edu.cn (Y.M.); guoxiuyun@ecupl.edu.cn (X.G.); 2023000108@ecupl.edu.cn (Z.C.); 2 Shanghai Jiao Tong University-Yale University Joint Center for Health Policy, Shanghai Jiao Tong University, Shanghai 200030, China; 3 School of Medicine, Shanghai Jiao Tong University, Shanghai 200025, China

**Keywords:** COVID-19, healthcare workers, monitoring mechanism, containment

## Abstract

The COVID-19 pandemic has exposed healthcare workers (HCWs) to serious infection risks. In this context, the proactive monitoring of HCWs is the first step toward reducing intrahospital transmissions and safeguarding the HCW population, as well as reflecting the preparedness and response of the healthcare system. As such, this study systematically reviewed the literature on evidence-based effective monitoring measures for HCWs during the COVID-19 pandemic. This was followed by a meta-synthesis to compile the key findings, thus, providing a clearer overall understanding of the subject. Effective monitoring measures of syndromic surveillance, testing, contact tracing, and exposure management are distilled and further integrated to create a whole-process monitoring workflow framework. Taken together, a mechanism for the early detection and containment of HCW infections is, thus, constituted, providing a composite set of practical recommendations to healthcare facility leadership and policy makers to reduce nosocomial transmission rates while maintaining adequate staff for medical services. In this regard, our study paves the way for future studies aimed at strengthening surveillance capacities and upgrading public health system resilience, in order to respond more efficiently to future pandemic threats.

## 1. Introduction

COVID-19 is caused by the transmission of severe acute respiratory syndrome coronavirus 2 (SARS-CoV-2), which has ravaged nations across the globe since late 2019 [1]. As of 29 April 2022, there were more than 510 million confirmed cases worldwide [2]. The rate of infection continues to rise, with emerging variants eroding mankind’s progress in combatting COVID-19. In this context, a wide range of doctors, nurses, health professionals, administrators, and healthcare staff have played crucial roles in the fight [3]. As these healthcare workers (HCWs) have undertaken the responsibility of caring for a continually rising number of COVID-19 patients, they are essential for ensuring an effective response to the ongoing public health crisis.

Due to their special work environment, HCWs tend to be at a higher risk of contracting SARS-CoV-2 than the general population [4]. In view of the bidirectional nature of HCW infections, in which they contract the disease at work and then introduce it to the community, or vice versa [5], it is critical to proactively monitor HCW infections and prevent the HCW population from becoming a transmission hub [6]. Moreover, during an infectious disease outbreak, HCWs are a sentinel surveillance population. Effective monitoring of HCWs is one of the most important measures, not only enabling the prevention of onward transmission, but also reflecting the preparedness and response of the healthcare system [7].

Effective monitoring, including syndromic surveillance, testing, contact tracing, and exposure management, allows for the early detection and containment of potential clusters of infection, and curbs transmissions, both in the hospital setting and throughout the community at large [8,9,10]. Previous research has shown that syndromic surveillance among HCWs allows for the timely implementation of infection prevention and control (IPC) practices [11,12]. A combination of body temperature and acute respiratory illness monitoring is usually deemed an effective approach to syndromic surveillance [6], with some scholars suggesting that anosmia should be included as a COVID-19-related symptom [13,14]. Efficient testing enables the rapid identification and isolation of infected HCWs, which not only prevents onward transmissions, but also ameliorates staff shortages due to unnecessary quarantines [15,16]. Nevertheless, researchers have also pointed out that aggressive contact tracing usually provides a greater yield than mass testing [17,18]. Based on the specific contact scenario, the risk of exposure is subsequently assessed, so that appropriate measures can be taken accordingly [11]. In sum, effective contact tracing and exposure management are crucial for ensuring the timely detection of new infections, thus, preventing the continued spread of COVID-19.

Most previous studies have either focused on the introduction of individual monitoring measures or shared local experiences with processes, such as health surveillance and diagnostic evaluation among HCWs during the pandemic [5,6,11]. Meanwhile, few scholars have conducted in-depth analyses of these practices or systematically studied, from a whole-process perspective, what is the effective monitoring mechanism for detecting infections and securing health and safety for HCWs in the COVID-19 context. By extension, there is a lack of evidence for use in comparison and debate.

As such, this study aims to quest for the optimal HCW monitoring mechanism and provide practical recommendations for administrators of healthcare facilities, leadership of healthcare systems, as well as policy makers tackling this global issue. To ensure a comprehensive analysis of existing research findings and elevate them to a more coherent and synthesised corpus, we systematically reviewed the real-life practices of hospitals across the world, then selected and analysed successful experiences through a meta-synthesis of studies reporting on evidence-based effective monitoring approaches for HCWs in the COVID-19 context. Based on the results, we distilled a monitoring mechanism for the early detection and containment of HCW infections, including effective monitoring measures of syndromic surveillance, testing, contact tracing, and exposure management, and a whole-process workflow framework composed of these measures.

This study constitutes a pioneering effort to compile current knowledge on COVID-19 monitoring among HCWs, thus, providing a more comprehensive understanding of what is needed to effectively protect the HCW population while safeguarding the public throughout the pandemic. Our findings offer valuable information for health authorities who are updating national, regional, and local COVID-19 response plans while also providing a foundation for continued research into strengthened surveillance and increased public health system resilience. Ultimately, this will help ensure more efficient responses to current and future outbreaks of other acute respiratory infection.

## 2. Methods

### 2.1. Search Strategy and Selection Criteria

This review was conducted according to the Preferred Reporting Items for Systematic Reviews and Meta-Analyses (PRISMA) guidelines [19]. The PRISMA and MOOSE (Meta-analysis of Observational Studies in Epidemiology) checklists are provided as Supplementary Materials Files S1 and S2. We searched both the Web of Science and PubMed for relevant literature using predefined terms, including ‘COVID-19’ AND ‘healthcare workers’ OR ‘healthcare professionals’ OR ‘healthcare workforce’ OR ‘healthcare personnel’. Articles were eligible for inclusion in this review if they met the following criteria: (1) pertained to the evidence-based monitoring measures of syndromic surveillance, testing, contact tracing, and/or exposure management for HCWs during the COVID-19 pandemic; (2) discussed methods with proven effectiveness in the early detection and management of HCW exposures/infections and/or provided data to support the main viewpoints; and (3) written in English.

We took steps to ensure that the screening process was as comprehensive as possible. First, two independent researchers screened articles published between April 2020 and March 2022 based on their titles and abstracts. Second, the full texts of studies included after the first step were obtained and further scrutinised to assess their overall eligibility based on the selection criteria. Finally, a third researcher was consulted when the first two disagreed about the relevance of any given article.

Additionally, risk-of-bias assessment was performed by two independent researchers, with disagreements discussed through consensus meetings. The Risk-of-Bias in Non-randomized Studies of Interventions tool (ROBINS-I) was employed to assess the reliability and validity of the potentially included studies.

### 2.2. Data Analysis

Based on (1) WHO technical guidance for COVID-19 monitoring among HCWs, (2) our own basic exploration of previous studies, and (3) in-depth interviews with experts (described in the next subsection), we identified syndromic surveillance, testing, contact tracing, and exposure management as the key elements of an effective monitoring mechanism for the early detection and containment of HCW infections [7,8].

We extracted relevant data from articles that were deemed eligible based on the procedures described in the previous subsection. This included basic publication information (i.e., author(s), accepted month/year, and study locations), study type, monitoring measures (i.e., syndromic surveillance, testing, contact tracing, and exposure management), and results. We then conducted a meta-synthesis by compiling and connecting key findings, discussing major disagreements about certain measures, and distilling these elements into practical recommendations for the effective monitoring, management, and protection of HCWs during the pandemic.

### 2.3. In-Depth Interviews

We recruited three experts with whom we conducted in-depth interviews. At the time of research, Expert 1 was a doctor at the Fever Clinic (a unit affiliated with the Emergency Department, specialising in the screening of infectious diseases [20]) in a tertiary hospital in Shanghai, Expert 2 was a doctor working with the Department of Pathology at a top medical centre in New York City, and Expert 3 was a health policy professor at a top university in China who focused on public health emergency preparedness. All interviewees were asked to evaluate the importance of syndromic surveillance, testing, contact tracing, and exposure management in the context of an HCW monitoring mechanism. They were also asked whether any other crucial elements should be considered based on their real-life practices and/or research findings.

## 3. Results

### 3.1. Studies Included

**Figure 1:**
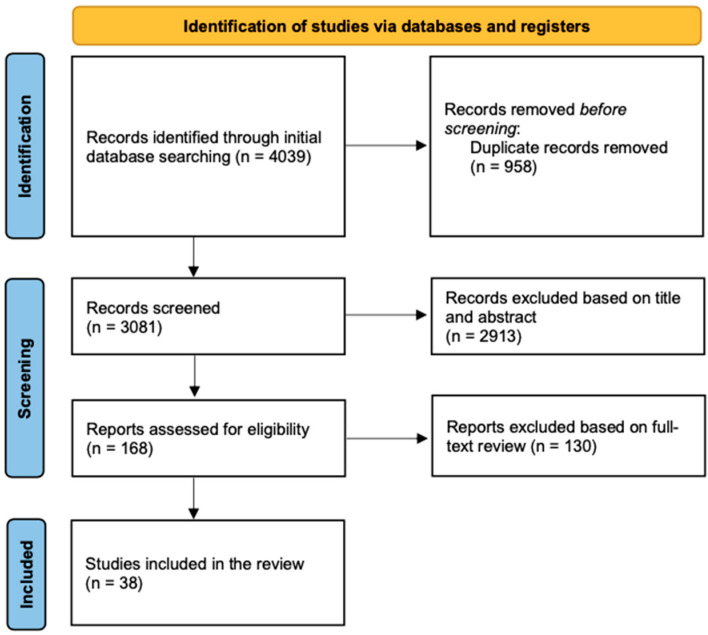
PRISMA flowchart showing the selection process.

After systematically searching the two databases, we initially included a total of 4039 articles. After removing duplicates, we screened 3081 articles based on their titles and abstracts. Of these, we retrieved the full texts of 168 for a full eligibility assessment. Ultimately, we included 38 articles in the final review (Figure 1).

### 3.2. Study Characteristics

**Figure 2:**
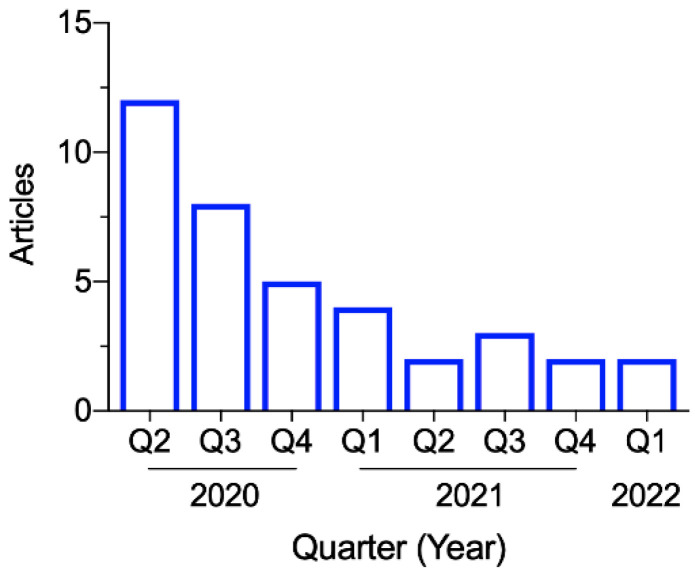
Included articles published between April 2020 and March 2022.

**Figure 3:**
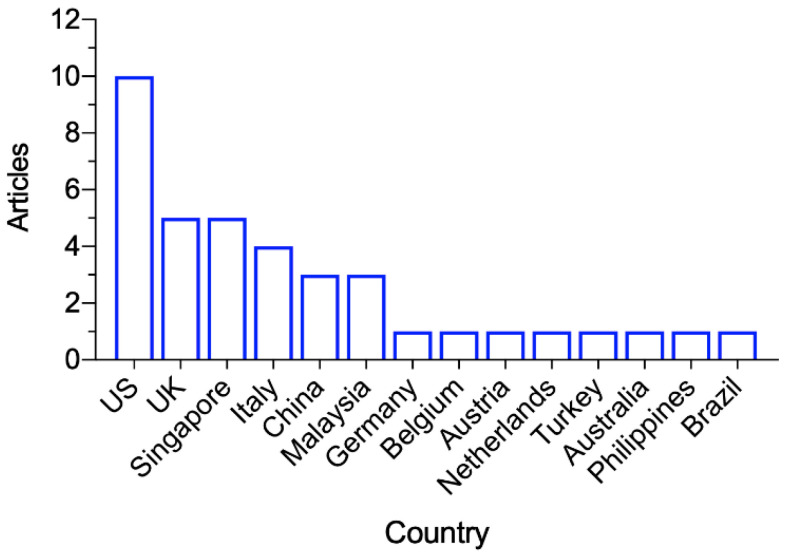
Study locations of the included articles.

The 38 articles were published between April 2020 and March 2022, with the most in the second quarter of 2020 (*n* = 12), during which the WHO dashboard showed a global surge of confirmed COVID-19 cases [2]. Figure 2 shows the publication dates for the included articles. As shown in Figure 3, the studies were conducted in the following countries: the US (*n* = 10), UK (*n* = 5), Singapore (*n* = 5), Italy (*n* = 4), China (*n* = 3), Malaysia (*n* = 3), Germany, Belgium, Austria, Netherlands, Turkey, Australia, Philippines, and Brazil (*n* = 1 from each).

Table 1 shows the main characteristics of the 38 studies, including the basic publication information, study type, relevant effective monitoring measures in the COVID-19 context (syndromic surveillance, testing, contact tracing and exposure management), and summary results. With duplicates (repeated count), 11 of the included studies discussed syndromic surveillance measures [6,11,21,22,23,24,25,26,27,28,29]; 28 studies discussed approaches of testing [3,6,11,22,23,26,27,29,30,31,32,33,34,35,36,37,38,39,40,41,42,43,44,45,46,47,48,49]; 16 studies discussed measures of contact tracing and exposure management [6,11,17,18,22,23,24,25,26,28,36,47,50,51,52,53].

### 3.3. Risk-of-Bias Assessment

Table 2 summarises the overall risk-of-bias assessment of the 38 studies. Most of the studies (*n* = 33) were assessed as low risk of bias, while five studies were considered moderate risk of bias. With duplicates (repeated count), 5 studies have low bias due to confounding factors, 23 studies have low bias in selection of participants, 28 studies have low bias in missing data and selection of the reported result, 35 studies have low bias in measurement of outcomes, and all studies have low bias in classification of interventions and deviations from intended interventions.

### 3.4. A Whole-Process Workflow Framework

**Figure 4:**
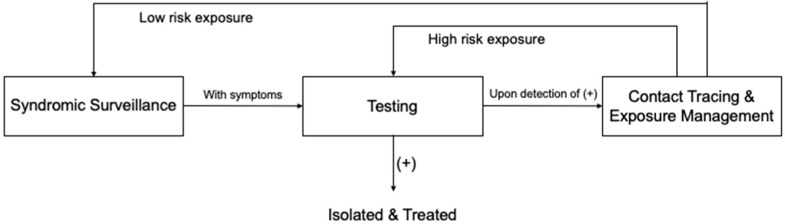
HCW monitoring workflow framework.

By synthesising the practices of the included studies, we constructed a whole-process HCW monitoring workflow framework, which can begin with either syndromic surveillance as a routine practice for HCWs or with testing when PCR tests for HCWs are conducted on a regular basis (Figure 4).

Syndromic surveillance facilitates the early detection of COVID-19-related symptoms. Upon onset of symptoms, HCWs are generally required to report to their relevant departments for testing. In some cases, symptomatic HCWs will initially be given medical leave for a five-day home quarantine; if symptoms continue, they will then be appointed for testing [22]. Once a positive case is, thus, identified, the case is isolated and treated. Meanwhile, contact tracing is initiated [52]. During this process, information will be obtained and collated on the individuals who have been in contact with the index case. Accordingly, identified contacts will be assessed for exposure risk and, thus, stratified based on the contact scenarios [52]. Those with high-exposure risk will be tested immediately, while those with low-exposure risk may be placed under daily syndromic surveillance for a period lasting 14 days after their most recent exposure [10]. Any contacts who present symptoms while under syndromic surveillance will be referred for testing. New rounds of contact tracing and exposure management will begin in cases where contacts are, thus, confirmed to have SARS-CoV-2 infections.

#### 3.4.1. Syndromic Surveillance

Compared with health authorities assessing HCWs’ symptoms on a daily basis, self-monitoring and reporting are found to be more feasible and efficient, especially during the exponential phase of a pandemic [5]. More specifically, HCWs should be instructed to measure their temperatures each day and report to their HCW surveillance teams if presenting a fever or any respiratory symptoms. Yombi et al. found that fever had a positive impact on the yield of PCR for SARS-CoV-2 (*p* < 0.001), utilizing fever as a selection criterion, resulting in more efficient screening [34]. Furthermore, scholars recommend immediate reporting, with low-threshold access being crucial given that some HCWs are reluctant to report mild symptoms due to concerns about burdening the system [5,22].

Comprehensive e-surveillance systems, web-based self-service applications, and online databases have been developed to facilitate reporting. Empirical data show that these digital tools are highly effective [21,24,26]; in this regard, they allow healthcare workers to easily and efficiently report their daily temperatures and/or any COVID-19-related symptoms via mobile device. The same online platforms can also be used to schedule testing appointments, redistribute workforces, and assist in epidemiological investigations [24,54]. All relevant data and other information are documented within these systems, thus, allowing hospital surveillance groups and outbreak management teams to track the wellbeing of HCWs, in addition to analysing trends that may help determine whether potential infection clusters are imminent.

#### 3.4.2. Testing

While studies from across the globe assert that HCWs should be given low-threshold access to testing [5,54], there is still ardent debate on whether asymptomatic workers should be provided with comprehensive testing in all cases. Some scholars support universal testing for HCWs, regardless of the symptoms [15,31,35]. Khalil et al. emphasised that mass testing allows for the early detection of asymptomatic infected HCWs, which can greatly reduce the risk of nosocomial transmission [32]. Treibel et al. also suggested that asymptomatic HCWs should be given easy access to testing, especially during new waves of infection [3,55]. Nevertheless, healthcare systems are typically under enormous pressures during any outbreak, in which case, such provisions are much more limited, especially during exponential phases and when time and/or resources are scarce. Meanwhile, studies have shown that symptoms are the best predictors of SARS-CoV-2 infections, with some scholars, thus, pointing out that it is not necessary to test asymptomatic HCWs who work in hospitals with sufficient PPE supplies and effective IPC measures [24,25,30,56,57]. Further, negative testing results cannot completely exclude infection [18]. The practice of testing asymptomatic HCWs not only entails the disadvantage of requiring frequent evaluations because intermittent testing may not capture asymptomatic infections, but may also lead to false negatives for exposed HCWs who are supposed to be placed under quarantine [5].

In the early phases of the COVID-19 pandemic, serological antibody testing was usually used in combination with RT-PCR (reverse transcription-polymerase chain reaction) testing to enhance efficiency of HCW screening [38,40]. While RT-PCR testing demonstrates active infections, serological testing reflects COVID-19 prevalence [39]. Studies have shown that even though the reliability of serological testing needs further validation, it is a useful screening tool for assessing the infection seroprevalence and is informative on infection susceptibility [29,37]. It is reported that parallel orthogonal testing for total SARS-CoV-2 antibodies using a commercial antibody detecting system and Enzyme-linked immunosorbent assay (ELISA) have been shown to improve the predictive value of serological tests [43]. Nevertheless, with vaccination rates increasing, serological antibody testing is no longer applicable, since it can hardly identify whether the humoral immune response is caused by viral infection or vaccination. On the contrary, the RT-PCR test, with excellent sensitivity and specificity, has been considered the “gold standard” for COVID-19 diagnosis, by which the cycle threshold (Ct) number is correlated with the estimated viral load [41]. Different RT-PCR testing approaches for HCWs, such as test–retest strategy [35], rostered routine testing [48] and a drive-through testing model [33], have been raised and demonstrated to be useful in detecting HCW infection and guiding HCWs to a safe return to duty.

**Figure 5:**
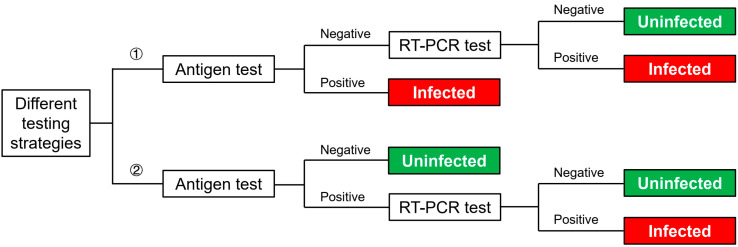
Different testing strategies.

As the pandemic has continued for more than two years and continues to bring challenges to healthcare systems across the globe, antigen testing has been developed in response to the urgent need for rapid and visualized diagnoses of SARS-CoV-2. Despite its relatively lower sensitivity compared with RT-PCR due to methodological reasons, the antigen test has unique advantages, such as a short testing time of up to 15 min, and independent to equipment and trained professionals to interpret the results. According to the current CDC recommendation, the frequency of the antigen test can be considered as a break-controlling measure [58]. Additionally, it has been reported that compared with traditional measures of placing exposed HCWs under 14-day quarantine, antigen tests for those HCWs every other day will reduce the total cost by 87% [27]. On the other hand, antigen tests have been widely used for the screening of at-risk populations, together with a follow-up RT-PCR test for confirmation, which greatly improves the testing efficiency [45]. An effective strategy is to provide antigen tests for HCWs first, and (1) if tested with positive antigen results, they would be considered SARS-CoV-2 positive; or (2) if tested with negative antigen results, they would be provided with further PCR tests for confirmation [45]. Another strategy would be providing follow-up PCR tests only for those tested with positive antigen results (Figure 5) [46]. According to the CDC guideline, the specificity of the antigen test is comparable to the RT-PCR test, which means false-positive results are unlikely [58]. Thus, Kolwijck’s testing strategy is more rational as, in this way, PCR testing better compensates antigen testing’s inadequate detection limit.

#### 3.4.3. Contact Tracing and Exposure Management

Contact tracing should be conducted upon the detection of a positive SARS-CoV-2 case [22,59]. A widely accepted approach is to interview the index case to collect information and gather listings of close contacts, as supplemented with information from the hospital’s medical records, thus, tracking healthcare processes and identifying other HCWs/patients with whom the infected case has interacted during periods of infectivity [11]. Hong et al. found that utilizing EHR clinical event data along with traditional methods would enhance the yield of contacts, with an increase of 22.2% that would have been neglected [28]. Although different countries/regions vary slightly in their applied definitions, a close contact commonly refers to a person who has been exposed to the index case within a distance of two meters for a duration of more than 15 min, up to two days prior to the onset of symptoms (or for asymptomatic infections, two days prior to collecting the positive sample) [10,60,61]. Nevertheless, Coppeta et al. evaluated the infection rate of HCWs in relation to determinants of exposure, surprisingly finding that only mask usage had significant effects on the chance of contagion (*p* < 0.01), and neither close-distance (within two meters) contact with an infected case, nor exposure for a duration of over 15 min was a significant factor [25]. This indicates that guidelines and recommendations constantly require modification in response to new evidence.

Risk of exposure is assessed based on the specific contact scenario, including the use of PPE/adherence to the IPC measures, and the type of occupational exposure [47]. All identified contacts are usually categorised into different risk groups so that measures can be taken accordingly [36,62]. Contacts presenting symptoms are considered at high risk and should be tested immediately, while those with low risk of exposure (i.e., presenting no symptoms and exposed for less than 15 min, at a distance of up to two meters while using proper PPE) are allowed to continue working, but may require daily health surveillance [22,23]. Additionally, at some hospitals, those having participated in aerosol-generating procedures for infected patients without proper PPE, regardless of presenting symptoms or not, are also classified in the high-risk group, and testing as well as quarantine are required [53].

Moreover, emerging technologies have been developed to enhance the accuracy and efficiency of both contact tracing and exposure management. This includes real-time location systems (RTLS), by which individuals wearing RTLS tags can be located within a certain premise, and closed-circuit television (CCTV) footage, which provides visual aids. Both have been found to enhance sensitivity and specificity if combined with conventional methods for extracting data from clinical databases [50,51,63]. Tracing applications are also useful ways to enhance the reliability of contact identification [64], while the analysis of big data platforms can help researchers quickly detect COVID-19 ‘hot spots’ [65].

## 4. Discussion

As a sentinel surveillance population, the rise of HCW infection rates reflects the spread of infection among the overall population [66]. This study made pioneering efforts in its exploration of an effective monitoring mechanism for HCWs during the COVID-19 pandemic. By synthesising evidence from the current literature, we provided a clear set of practical recommendations for more effectively monitoring and safeguarding the healthcare workforce. In addition to the above description of effective measures and whole-process workflow framework, the main findings from the meta-synthesis are further discussed, striving for the optimal mechanism for both mitigating the risks of nosocomial transmission and maintaining adequate staff for medical services.

Our study is also consistent with previous research, regarding prevention measures for other acute respiratory infections, such as influenza and severe acute respiratory syndrome (SARS) [67]. Since the viruses accounting for acute respiratory infections have the similar mode of transmission and testing methods, our study paves the way for future studies aimed at strengthening surveillance capacities and upgrading public health system resilience, in order to respond more efficiently to future threats of other acute respiratory infections.

### 4.1. Future Directions

By reviewing the included articles, we also found that a sophisticated monitoring mechanism would be even more effective for promptly detecting outbreaks, if supported by a comprehensive outbreak management strategy, which is usually led by a multidisciplinary team that monitors all activities throughout the process. This involves the collection and collation of data related to the status of HCWs, thus, providing a robust way to analyse trends so that potential infection clusters can be identified at an early stage and, in turn, appropriate containment practices can quickly be implemented [6,11,22]. Future research may add to these findings by investigating effective outbreak management measures and assessing how they can be synchronised with the HCW monitoring workflow framework provided herein.

Additionally, we found that IoT and AI enhanced smart administrations in various areas, including the early warning of new infection waves, real-time situational surveillance, and optimal resource allocation [68,69]. As such, HCW infections can be more efficiently monitored and contained with the help of these emerging technologies. Future studies should explore their application in syndromic surveillance, testing, contact tracing, and exposure management.

### 4.2. Limitations

This study also had some limitations. First, only three experts were recruited for the in-depth interviews. However, we contend that their experience was truly valuable, and recommend that future studies include more frontline professionals, thus, providing a more comprehensive perspective on the most effective methods for monitoring, managing, and protecting HCWs, through optimal resource usage. Regarding the assessment of exposure risks, the literature also shows most practices are currently based on a combination of official guidelines and informal rules, both of which require further examination.

## 5. Conclusions

COVID-19 has created daunting challenges for people across the globe. As HCWs play indispensable roles in combating this crisis, it is critically important to provide them with adequate protection. In turn, this ensures continued medical care for patients while limiting viral spread. A major first step toward achieving this is to ensure effective monitoring for HCWs.

Based on a systematic review and meta-synthesis of the current literature, this study analysed prominent areas of ongoing debate and distilled a mechanism for the early detection and containment of infections among HCWs, with effective measures, including syndromic surveillance, testing, contact tracing, and exposure management. To guide this, we also constructed a whole-process workflow framework.

The effective monitoring mechanism offers a composite set of practical recommendations for healthcare facility administrators and policy makers, which are valuable for continued research into strengthened surveillance and increased public health system resilience. This will also help ensure more efficient responses to future threats of other acute respiratory infection outbreaks.

## Figures and Tables

**Table 1:** Study characteristics.

No.	Study Characteristics and Summary Report	
1	Author	Zhang et al. [21]
	Month/Year	April 2020
	Country	US
	Study Type	Observational study
	Measures	Syndromic Surveillance: A web-based mobile responsive HCW symptom screening application
	Results	Over a 7-day period, having quickly identified 0.36% symptomatic HCWs that otherwise could have come to work, increasing efficiency and effectiveness
2	Author	Hunter et al. [30]
	Month/Year	April 2020
	Country	UK
	Study Type	Observational study
	Measures	Testing: Testing of staff with compatible symptoms and conveying results rapidly via email
	Results	In 3 weeks, enabled 1414 (out of 1654) HCWs to return more rapidly to service
3	Author	Treibel et al. [3]
	Month/Year	May 2020
	Country	UK
	Study Type	Observational study
	Measures	Testing: Testing the asymptomatic HCWs especially during potential new waves of infection
	Results	Asymptomatic HCWs should be given easy access to testing, especially during new waves of infection
4	Author	Wee et al. [22]
	Month/Year	May 2020
	Country	Singapore
	Study Type	Observational study
	Measures	Syndromic Surveillance: Ongoing syndromic surveillance and centralized reporting of fever and ARI symptoms Testing: Testing the symptomatic HCWs if symptoms not resolve after 5 days Contact Tracing & Exposure Management: (1) Contact tracing conducted upon detection of a confirmed case; (2) Exposure risk assessment based on duration of contact, type of activity, and PPE use during the contact; (3) To test the exposed HCWs developing symptoms; to quarantine HCWs having significant unprotected exposure; to active monitor symptoms of the HCWs with low risk of exposure;
	Results	Over a 16-week period, 14 cases of HCW infection and 4 clusters detected After measures taken, zero nosocomial transmission detected Early detection having reduced quarantine of HCWs
5	Author	Garzaro et al. [23]
	Month/Year	May 2020
	Country	Italy
	Study Type	Observational Study
	Measures	Syndromic Surveillance: HCWs identified as low risk of exposure to self-monitor symptoms including cough, fever, dyspnoea, anosmia; Testing: Early testing enabling faster return-to-work thus alleviating staff shortages; Contact Tracing & Exposure Management: (1) Fast identification of contacts with the infected critical to lowering nosocomial transmission; (2) A structured risk-management for HCW exposure: (i) stratifying risks into high risk: presenting symptoms; moderate risk: exposure >15 min, or <2 m, without PPE; low risk: <15 min, or >2 m, with PPE; (ii) high risk HCWs to get tested and home quarantined; moderate risk HCWs to use surgical masks while awaiting the test results; low risk HCWs to self-monitor symptoms;
	Results	The monitoring measures having significantly reduced time between exposure, warning, and testing (*p* < 0.001)
6	Author	Rivett et al. [31]
	Month/Year	May 2020
	Country	UK
	Study Type	Observational Study
	Measures	Testing: Comprehensive testing of both symptomatic & asymptomatic HCWs
	Results	Data suggesting the true asymptomatic carriage rate being 0.5% Comprehensive testing of HCWs with minimal/no symptoms critical for protecting HCWs and patients
7	Author	Khalil et al. [32]
	Month/Year (Accepted)	May 2020
	Country	UK
	Study Type	Observational Study
	Measures	Testing: Universal testing of HCWs
	Results	34% positive HCW cases being asymptomatic while 59% symptomatic HCWs tested negative, indicating crucial needs for routine testing of all HCWs to (1) identify asymptomatic infected HCWs in an early stage, and (2) mitigate staff shortages due to unnecessary quarantine
8	Author	Flynn et al. [33]
	Month/Year	May 2020
	Country	US
	Study Type	Observational Study
	Measures	Testing: A drive-through testing model
	Results	The drive-through testing model having increased test efficiency, avoided long lines, conserved PPE
9	Author	Buchtele et al. [18]
	Month/Year	May 2020
	Country	Austria
	Study Type	Observational Study
	Measures	Contact Tracing & Exposure Management: Extensive contact tracing implemented among HCWs caring for immunocompromised patients, with all those having face-to-face contact with the confirmed case since the case’s onset of symptoms to get tested regardless of length of exposure
	Results	Extensive contact tracing and mass testing having prevented further spread of nosocomial transmission
10	Author	Ho et al. [50]
	Month/Year	May 2020
	Country	Singapore
	Study Type	Observational Study
	Measures	Contact Tracing & Exposure Management: RTLS-based (real-time location systems) contact tracing demonstrated having better validity than traditional EMR-based (electronic medical record) methods;
	Results	An integration of RTLS and EMR providing the best performance for contact tracing with a sensitivity of 77.8% and a specificity of 73.4%
11	Author	Yombi et al. [34]
	Month/Year	May 2020
	Country	Belgium
	Study Type	Observational Study
	Measures	Testing: Fever as a criterion for testing
	Results	Fever having a positive impact on the yield of PCR for SARS-CoV-2 (*p* < 0.001), using fever as a selection criterion resulting in more efficient screening
12	Author	Blain et al. [35]
	Month/Year	June 2020
	Country	US
	Study Type	Observational Study
	Measures	Testing: A test-retest strategy
	Results	11% asymptomatic HCWs with negative PCR results developing antibodies later in time Repeated testing effective in identifying asymptomatic infected HCWs
13	Author	Wang et al. [24]
	Month/Year	July 2020
	Country	Singapore
	Study Type	Observational Study
	Measures	Syndromic Surveillance: A comprehensive HCW sickness surveillance system: online reporting platform, medical screening, and testing for all the symptomatic HCWs Contact Tracing & Exposure Management: Exposure factors: serving in COVID-19 area/in non-COVID-19 area with known close contacts/in non-COVID-19 area with no known close contacts
	Results	Despite enhanced monitoring mechanism, no HCW was identified with infection, suggesting universal testing of HCWs not necessary for hospitals with adequate PPE protocol
14	Author	Villanueva et al. [36]
	Month/Year	July 2020
	Country	Philippines
	Study Type	Observational Study
	Measures	Testing: Criteria for testing: close contact with or high-risk exposure to a COVID-19 case, presence of symptoms Contact Tracing & Exposure Management: Categorizing exposure into high/medium/low risks based on duration of contact, PPE use, whether an aerosol generating procedure
	Results	Early screening for HCW infection having reduced nosocomial transmission
15	Author	Mehta et al. [17]
	Month/Year	July 2020
	Country	US
	Study Type	Observational Study
	Measures	Contact Tracing & Exposure Management: Aggressive contact tracing enabling the identification & monitoring of asymptomatic and/or pre-symptomatic HCWs
	Results	Aggressive and effective contact tracing providing greater yield than mass testing of every individual
16	Author	Kacmaz et al. [37]
	Month/Year	August 2020
	Country	Turkey
	Study Type	Observational Study
	Measures	Testing: rapid antibody testing
	Results	Reliability of antibody testing needing further validation but useful in COVID-19 screening among HCWs to evaluate IPC measures and prevent intra-hospital infection
17	Author	Tong et al. [38]
	Month/Year	August 2020
	Country	China (Mainland)
	Study Type	Observational Study
	Measures	Testing: A combination of PCR testing, serological testing, and radiological assessment conducted among HCWs caring for COVID-19 patients in the early stage of the outbreak
	Results	With the measures taken, no nosocomial infection detected
18	Author	Racine-Brzostek et al. [39]
	Month/Year	September 2020
	Country	US
	Study Type	Observational Study
	Measures	Testing: PCR + antibody testing
	Results	100% PCR positive HCWs tested positive for antibody testing High rates of seroprevalence suggesting the need for expanded PCR testing for HCWs
19	Author	Del Castillo et al. [40]
	Month/Year	September 2020
	Country	Italy
	Study Type	Observational Study
	Measures	Testing: Serological testing followed by PCR testing if positive to IgG
	Results	Serological IgG testing combined with PCR testing found to be a valid screening intervention
20	Author	Ho et al. [51]
	Month/Year	September 2020
	Country	Singapore
	Study Type	Observational Study
	Measures	Contact Tracing & Exposure Management: Utility of surveillance technologies such as RTLS and CCTV systems to enhance HCW exposure management
	Results	Having objectively identified 55 HCWs with 7 prolonged exposures (≥30 min), enabling more effective contact tracing than traditional methods
21	Author	Chong et al. [11]
	Month/Year	October 2020
	Country	Malaysia
	Study Type	Observational Study
	Measures	Syndromic Surveillance: HCWs with identifiable exposure risk under daily syndromic surveillance (self-assessment and self-reporting of symptoms through an online system) for 14 days since last exposure to an infection Testing: Targeted testing of close contacts Contact Tracing & Exposure Management: (1) Intensive contact tracing with identified close contacts having their exposure assessed and grouped into high/medium/low risk based on duration of exposure, presence of symptoms, PPE use, and whether an aerosol-generating procedure; (2) All close contacts to get tested and under daily symptom surveillance for 14 days; (3) HCWs with high risk exposure to be quarantined for 14 days; with medium risk 7 days; with low risk 2 days of sick leave
	Results	In a period of 5 months, 2401 risk assessments carried out among 1408 HCWs The surveillance program having limited nosocomial transmission, with a cumulative incidence of HCW infection of 0.3%
22	Author	Chen et al. [6]
	Month/Year	November 2020
	Country	China (Taiwan)
	Study Type	Observational Study
	Measures	Syndromic Surveillance: Centralized reporting of fever and ARI symptoms Testing: Testing the symptomatic Contact Tracing & Exposure Management: HCW exposure history reporting system
	Results	With the measures taken, no HCW infection detected
23	Author	Domeracki et al. [41]
	Month/Year	November 2020
	Country	US
	Study Type	Observational Study
	Measures	Testing: PCR cycle threshold (Ct) data used for HCW return to work (RTW) decisions
	Results	Initial Ct data significantly correlated with the time period between first diagnosis and RTW clearance (r = −0.80, *p* < 0.01), supplementing the dichotomized positive-or-negative PCR results
24	Author	Buising et al. [42]
	Month/Year	November 2020
	Country	Australia
	Study Type	Observational Study
	Measures	Testing: Frequent testing of HCWs and patients in wards with outbreaks and quick turnaround time for test results
	Results	Rapid and accessible testing enabling real-time outbreak management
25	Author	Coppeta et al. [25]
	Month/Year	December 2020
	Country	Italy
	Study Type	Observational Study
	Measures	Syndromic Surveillance: Exposed HCWs placed under an active syndromic surveillance program Contact Tracing & Exposure Management: Evaluating (1) distance from the infected, (2) duration of exposure, (3) the kind of medical service provided during the exposure, and (4) use of PPE
	Results	Typical symptoms presented in 92% HCW positive cases, but in only 33.3% negative cases (*p* < 0.01), suggesting symptoms being the best predictors of positive PCR results Close contact (within 2 m for more than 15 min) not statistically connected to contagion Use of mask significantly related to contagion (*p* < 0.01)
26	Author	Mullins et al. [43]
	Month/Year	January 2021
	Country	US
	Study Type	Experimental Study
	Measures	Testing: Parallel orthogonal testing of (1) Ortho Vitros Test, a commercial immunodiagnostic system, and (2) UMMC ELISA, a manually developed ELISA for total SARS-CoV-2 antibodies and full-length spike ectodomain protein
	Results	Positive predictive value: Ortho Vitros (82.2%), UMMC ELISA (100%) Negative predictive value: Ortho Vitros (100%), UMMC ELISA (99.9%) Parallel orthogonal testing of both demonstrated to improve the predictive value (+: 100%, −: 100%)
27	Author	Cheng et al. [26]
	Month/Year	March 2021
	Country	China (Hong Kong)
	Study Type	Observational Study
	Measures	Syndromic Surveillance: electronic syndromic surveillance system activated since the 1st imported case Testing: (1) PCR testing for symptomatic HCWs and HCWs classified as close contacts; (2) Repeated testing according to clinical assessment Contact Tracing & Exposure Management: (1) infection control team leading epidemiological investigation; (2) classifying the infected into hospital-acquired, community-acquired, and undetermined
	Results	Infection rate of HCWs (0.46‰) significantly lower than that of general population (0.71‰) (*p* < 0.01) No nosocomial transmission detected among HCWs
28	Author	Monsalud et al. [53]
	Month/Year	March 2021
	Country	US
	Study Type	Observational Study
	Measures	Contact Tracing & Exposure Management: (1) high-risk exposure HCWs (having participated in aerosol-generating procedures without adequate PPE; ongoing exposure to infected household members) required to self-quarantine and PCR testing; (2) low-risk exposure HCWs (all the other exposed HCWs) placed under surveillance
	Results	7.6% low-risk exposure HCWs identified as PCR-positive
29	Author	Wan et al. [52]
	Month/Year	March 2021
	Country	Malaysia
	Study Type	Observational Study
	Measures	Contact Tracing & Exposure Management: (1) contact tracing initiated once a COVID-19 case identified, collating info on the movement of the case 48 h before the onset of symptoms/diagnosis, forming a list of contacts; (2) level of risk of the contacts assessed and classified into different groups; (3) detailing management algorithm for low/medium/high-risk HCWs
	Results	Risk-based assessment with high sensitivity (100%) and specificity (72%) Risk categories and symptoms significantly correlated with positive cases (*p* < 0.001)
30	Author	Fernandes et al. [44]
	Month/Year	April 2021
	Country	Brazil
	Study Type	Observational Study
	Measures	Testing: PCR testing for the symptomatic HCWs and, if negative, a 2nd PCR test after the 5th day since symptom onset
	Results	The 2nd PCR testing having detected 4.9% of the positive cases
31	Author	Kolwijck et al. [45]
	Month/Year	April 2021
	Country	The Netherlands
	Study Type	Observational Study
	Measures	Testing: Antigen test for symptomatic HCWs, and (1) if tested positive, considered COVID-19 infection; (2) if tested negative, followed by PCR testing
	Results	The antigen-based testing strategy proved to be effective and easy to implement, with 72.5% sensitivity and 97% negative predictive value
32	Author	Lamb et al. [46]
	Month/Year	July 2021
	Country	UK
	Study Type	Observational Study
	Measures	Testing: Mass antigen testing for HCWs, followed by PCR testing if antigen tested positive
	Results	Antigen testing proven to be an effective screening tool, with a positive predictive value of 94.21%
33	Author	Azami et al. [47]
	Month/Year	July 2021
	Country	Malaysia
	Study Type	Observational Study
	Measures	Testing: PCR + serological testing Contact Tracing & Exposure Management: (1) Online questionnaire; (2) Evaluating risk based on HCWs’ occupational exposure and adherence to IPC practices
	Results	With measures taken, nosocomial infection having reduced, with an HCW infection rate of 0.5%
34	Author	Wee et al. [48]
	Month/Year	August 2021
	Country	Singapore
	Study Type	Observational Study
	Measures	Testing: Rostered routine testing for HCWs + mass screening of all inpatients
	Results	Enhancing early identification and contact tracing for HCW cases Significantly reducing the time infected inpatients spent in the general ward prior to isolation (*p* < 0.01)
35	Author	Diel et al. [27]
	Month/Year	October 2021
	Country	Germany
	Study Type	Observational Study
	Measures	Syndromic Surveillance: Exposed HCWs required to self-observe COVID-19-related symptoms Testing: Antigen testing every other day for exposed HCWs + additional PCR testing if one becoming symptomatic
	Results	Monitoring exposed HCWs with the measures in this study greatly reducing costs by 87.0%, compared with sending the exposed HCWs into quarantine
36	Author	Hong et al. [28]
	Month/Year	October 2021
	Country	US
	Study Type	Observational Study
	Measures	Syndromic Surveillance: HCWs confirmed with exposure registered for twice-a-day symptom monitoring for 14 days via email Contact Tracing & Exposure Management: Using electronic health record clinical event data (EHR report), in addition to traditional interviews, staff records, radio-frequency identification data, wifi access logs, bluetooth data, and etc. to enhance contact screening
	Results	22.2% exposures detected by EHR report, which would have been neglected based on traditional contact tracing methods
37	Author	Cordioli et al. [29]
	Month/Year	February 2022
	Country	Italy
	Study Type	Observational Study
	Measures	Syndromic Surveillance: Monitoring COVID-19 pathognomonic signs and symptoms Testing: Serological + PCR testing
	Results	Using a 3-diagnostic criterion (PCR + serological testing + pathognomonic presentation) to assess infection prevalence: COVID-19 prevalence varied based on different criterion: serological (6.7%), PCR (8.1%), serological/PCR (10.0%), pathognomonic presentation (9.6%), at least one of the above-mentioned criteria (17.6%) The probability of positive serological result decreasing by 1.1% every 10 days from the infection Data suggesting serological testing informative on infection susceptibility but not best for predicting previous infection
38	Author	Tande et al. [49]
	Month/Year	March2022
	Country	US
	Study Type	Observational Study
	Measures	Testing: Rapid antigen test for infected HCWs who meet the criteria to return to work, on the 5th day (or later) since symptom onset/diagnosis of COVID-19
	Results	The rapid antigen test, helpful to guide return-to-work decisions, having reduced isolation time by 2 days/person

**Table 2:** Risk-of-bias assessment.

Author and Year	Bias Due to Confounding	Bias in Selection of Participants into the Study	Bias in Classification of Interventions	Bias Due to Deviations from Intended Interventions	Bias Due to Missing Data	Bias in Measurement of Outcomes	Bias in Selection of the Reported Result	Overall Risk of Bias
Zhang et al. [21] April 2020	Low	Low	Low	Low	Moderate	Low	Low	Low
Hunter et al. [30] April 2020	Moderate	Moderate	Low	Low	Moderate	Moderate	Low	Moderate
Treibel et al. [3] May 2020	Moderate	Moderate	Low	Low	Low	Low	Moderate	Moderate
Wee et al. [22] May 2020	Moderate	Low	Low	Low	Low	Low	Low	Low
Garzaro et al. [23] May 2020	Low	Low	Low	Low	Low	Low	Low	Low
Rivett et al. [31] May 2020	Moderate	Low	Low	Low	Low	Low	Low	Low
Khalil et al. [32] May 2020	Moderate	Low	Low	Low	Moderate	Low	Low	Low
Flynn et al. [33] May 2020	Moderate	Low	Low	Low	Low	Low	Low	Low
Buchtele et al. [18] May 2020	Moderate	Moderate	Low	Low	Low	Low	Low	Low
Ho et al. [50] May 2020	Moderate	Moderate	Low	Low	Moderate	Low	Low	Low
Yombi et al. [34] May 2020	Moderate	Moderate	Low	Low	Moderate	Low	Moderate	Moderate
Blain et al. [35] June 2020	Moderate	Low	Low	Low	Low	Low	Low	Low
Wang et al. [24] July 2020	Moderate	Low	Low	Low	Low	Low	Low	Low
Villanueva et al. [36] July 2020	Moderate	Moderate	Low	Low	Low	Low	Moderate	Low
Mehta et al. [17] July 2020	Moderate	Moderate	Low	Low	Low	Low	Moderate	Moderate
Kacmaz et al. [37] August 2020	Moderate	Moderate	Low	Low	Low	Moderate	Low	Low
Tong et al. [38] August 2020	Moderate	Moderate	Low	Low	Low	Moderate	Low	Low
Racine-Brzostek et al. [39] September 2020	Moderate	Low	Low	Low	Low	Low	Low	Low
Del Castillo et al. [40] September 2020	Moderate	Low	Low	Low	Moderate	Low	Moderate	Low
Ho et al. [51] September 2020	Moderate	Moderate	Low	Low	Moderate	Low	Low	Low
Chong et al. [11] October 2020	Moderate	Moderate	Low	Low	Low	Low	Low	Low
Chen et al. [6] November 2020	Moderate	Low	Low	Low	Low	Low	Low	Low
Domeracki et al. [41] November 2020	Moderate	Moderate	Low	Low	Low	Low	Low	Low
Buising et al. [42] November 2020	Moderate	Low	Low	Low	Low	Low	Low	Low
Coppeta et al. [25] December 2020	Moderate	Low	Low	Low	Low	Low	Moderate	Low
Mullins et al. [43] January 2021	Low	Low	Low	Low	Low	Low	Low	Low
Cheng et al. [26] March 2021	Moderate	Low	Low	Low	Low	Low	Low	Low
Monsalud et al. [53] March 2021	Moderate	Moderate	Low	Low	Low	Low	Moderate	Low
Wan et al. [52] March 2021	Moderate	Low	Low	Low	Moderate	Low	Low	Low
Fernandes et al. [44] April 2021	Moderate	Moderate	Low	Low	Low	Low	Moderate	Moderate
Kolwijck et al. [45] April 2021	Moderate	Low	Low	Low	Low	Low	Low	Low
Lamb et al. [46] July 2021	Moderate	Low	Low	Low	Moderate	Low	Low	Low
Azami et al. [47] July 2021	Moderate	Moderate	Low	Low	Moderate	Low	Low	Low
Wee et al. [48] August 2021	Moderate	Moderate	Low	Low	Low	Low	Moderate	Low
Diel et al. [27] October 201	Low	Low	Low	Low	Low	Low	Low	Low
Hong et al. [28] October 2021	Moderate	Low	Low	Low	Low	Low	Moderate	Low
Cordioli et al. [29] February 2022	Low	Low	Low	Low	Low	Low	Low	Low
Tende et al. [49] March 2022	Moderate	Low	Low	Low	Low	Low	Low	Low

## Data Availability

Not applicable.

## Supplementary Materials

The following supporting information can be downloaded at: https://www.mdpi.com/article/10.3390/ijerph19105943/s1, File S1: PRISMA checklist, ref. [70] is cited in this file, File S2: MOOSE checklist.
