# Supplementary material for: An Effective Mechanism for the Early Detection and Containment of Healthcare Worker Infections in the Setting of the COVID-19 Pandemic: A Systematic Review and Meta-Synthesis
Source: Int J Environ Res Public Health. 2022 May 13;19(10):5943. doi: 10.3390/ijerph19105943 (PMC9141359; doi:10.3390/ijerph19105943)
Supplement: Supplementary file 1 [file ijerph-19-05943-s001.zip › Supplementary material-File S2_MOOSE_Checklist for ijerph-1731208.pdf]

MOOSE (Meta-analyses Of Observational Studies in Epidemiology) Checklist

A reporting checklist for Authors, Editors, and Reviewers of Meta-analyses of Observational Studies. You must report the page number in your manuscript where you consider each of the items listed in this checklist. If you have not included this information, either revise your manuscript accordingly before submitting or note N/A.

Reporting Criteria	Reported (Yes/No)	Reported on Page No.
**Reporting of Background**		
Problem definition	Yes	2
Hypothesis statement	No	
Description of Study Outcome(s)	Yes	2
Type of exposure or intervention used	No	
Type of study design used	Yes	3
Study population	Yes	1
**Reporting of Search Strategy**		
Qualifications of searchers (eg, librarians and investigators)	No	
Search strategy, including time period included in the synthesis and keywords	Yes	3
Effort to include all available studies, including contact with authors	Yes	3
Databases and registries searched	Yes	2
Search software used, name and version, including special features used (eg, explosion)	No	
Use of hand searching (eg, reference lists of obtained articles)	Yes	5
List of citations located and those excluded, including justification	Yes	3
Method for addressing articles published in languages other than English	Yes	3
Method of handling abstracts and unpublished studies	Yes	3
Description of any contact with authors	Yes	3
**Reporting of Methods**		
Description of relevance or appropriateness of studies assembled for assessing the hypothesis to be tested	Yes	3
Rationale for the selection and coding of data (eg, sound clinical principles or convenience)	Yes	3
Documentation of how data were classified and coded (eg, multiple raters, blinding, and interrater reliability)	Yes	3
Assessment of confounding (eg, comparability of cases and controls in studies where appropriate	Yes	13-15

Reporting Criteria	Reported (Yes/No)	Reported on Page No.
Assessment of study quality, including blinding of quality assessors; stratification or regression on possible predictors of study results	Yes	13-15
Assessment of heterogeneity	No	
Description of statistical methods (eg, complete description of fixed or random effects models, justification of whether the chosen models account for predictors of study results, dose-response models, or cumulative meta-analysis) in sufficient detail to be replicated	No	
Provision of appropriate tables and graphics	Yes	4-17
**Reporting of Results**		
Table giving descriptive information for each study included	Yes	5-12
Results of sensitivity testing (eg, subgroup analysis)	No	
Indication of statistical uncertainty of findings	No	
**Reporting of Discussion**		
Quantitative assessment of bias (eg, publication bias)	No	
Justification for exclusion (eg, exclusion of non–English-language citations)	Yes	3
Assessment of quality of included studies	Yes	13
**Reporting of Conclusions**		
Consideration of alternative explanations for observed results	No	
Generalization of the conclusions (ie, appropriate for the data presented and within the domain of the literature review)	Yes	18
Guidelines for future research	Yes	18
Disclosure of funding source	Yes	19

Once you have completed this checklist, please save a copy and upload it as part of your submission. DO NOT include this checklist as part of the main manuscript document. It must be uploaded as a separate file.
